# Epigenetic-Mediated Antimicrobial Resistance: Host versus Pathogen Epigenetic Alterations

**DOI:** 10.3390/antibiotics11060809

**Published:** 2022-06-16

**Authors:** Jibran Sualeh Muhammad, Naveed Ahmed Khan, Sutherland K. Maciver, Ahmad M. Alharbi, Hasan Alfahemi, Ruqaiyyah Siddiqui

**Affiliations:** 1 Department of Basic Medical Sciences, College of Medicine, University of Sharjah, Sharjah 27272, United Arab Emirates; nkhan@sharjah.ac.ae; 2 Centre for Discovery Brain Sciences, Edinburgh Medical School-Biomedical Sciences, University of Edinburgh, Edinburgh EH8 9XD, Scotland, UK; smaciver@exseed.ed.ac.uk; 3 Department of Clinical Laboratories Sciences, College of Applied Medical Sciences, Taif University, Taif 21944, Saudi Arabia; a.alharbii@tu.edu.sa; 4 Department of Medical Microbiology, Faculty of Medicine, Al-Baha University, P.O. Box 1988, Al-Baha 65799, Saudi Arabia; halfahmi@bu.edu.sa; 5 College of Arts and Sciences, American University of Sharjah, University City, Sharjah 26666, United Arab Emirates

**Keywords:** antibiotic resistance, epigenetic changes, DNA methylation, histone modifications, nucleoid-associated proteins, HU proteins

## Abstract

Since the discovery of antibiotics, humans have been benefiting from them by decreasing the morbidity and mortality associated with bacterial infections. However, in the past few decades, misuse of antibiotics has led to the emergence of bacterial infections resistant to multiple drugs, a significant health concern. Bacteria exposed to inappropriate levels of antibiotics lead to several genetic changes, enabling them to survive in the host and become more resistant. Despite the understanding and targeting of genetic-based biochemical changes in the bacteria, the increasing levels of antibiotic resistance are not under control. Many reports hint at the role of epigenetic modifications in the bacterial genome and host epigenetic reprogramming due to interaction with resistant pathogens. Epigenetic changes, such as the DNA-methylation-based regulation of bacterial mutation rates or bacteria-induced histone modification in human epithelial cells, facilitate its long-term survival. In this review article, epigenetic changes leading to the development of antibiotic resistance in clinically relevant bacteria are discussed. Additionally, recent lines of evidence focusing on human host epigenetic changes due to the human–pathogen interactions are presented. As genetic mechanisms cannot explain the transient nature of antimicrobial resistance, we believe that epigenetics may provide new frontiers in antimicrobial discovery.

## 1. Introduction

Microorganisms overexposed to antibiotics lead to antimicrobial resistance (AMR); hence, these microorganisms then emerge as long-term survivors. AMR poses an immense threat to public health in preventing and curing severe bacterial infections, leading to increased hospital length of stay and healthcare costs [1]. A predictive statistical model used by the Global Research on Antimicrobial resistance (GRAM) project estimated ~5 million deaths associated with AMR in 2019, indicating the substantial importance of preventing infections in the first place [2]. On the one hand, several classes of antibiotics have been discovered to target essential bacterial processes. On the other hand, evidence-based research has shown that microorganisms can develop sophisticated defense systems to survive in the host and become resistant to a range of antimicrobial agents, causing severe illness and death [3]. They are leading to an increase in AMR-induced mortality and morbidity and a worldwide spread of multi-drug-resistant (MDR), extensively drug-resistant (likelihood of being resistant to all, or almost all, approved antimicrobial agents), and pan-drug-resistant bacteria (resistant to all antimicrobial agents) [4,5].

Bacteria are the leading microorganisms that acquire drug resistance by utilizing multiple intrinsic or extrinsic mechanisms. Bacterial modifications leading to AMR are only one side of a two-faced coin. AMR mechanisms include the horizontal and vertical transfer of resistance genes, gene mutations affecting antibiotic targets, drug influx/efflux strategies, or antibiotic inactivation [6]. Among the common and severely affecting pathogens attributed to AMR development include *Escherichia coli*, *Staphylococcus aureus*, *Klebsiella pneumoniae*, *Mycobacterium tuberculosis*, *Helicobacter pylori*, and *Pseudomonas aeruginosa*, but there are many more [2]. In addition, previous reports have suggested that the host–pathogen interaction can lead to long-term immunological changes in the human host, causing improved survival of microorganisms [7,8]. In addition to the genetic mechanisms leading to AMR [9], there has been a sharp increase in research exploring the epigenetic-mediated AMR in bacteria and the human host. Since small molecular-sized epigenetic modifier drugs can reverse the epigenetic changes, they might have a strong potential to fight against resistant bacterial infections.

In this review article, for the first time, we present both perspectives—epigenetic changes in the host and the pathogen, which are responsible for mediating AMR in these species. Concerning the field of bacterial epigenetics, we provide details of the epigenetic changes and modulators leading to the development of antibiotic resistance in critical illness-causing bacteria. Additionally, we provide a comprehensive description of AMR due to the human host epigenome remodeling upon bacterial–host interactions. The potential role of epigenetic changes in developing strategies to diagnose, prevent, or treat resistant bacteria is also discussed. As genetic mechanisms are unable to thoroughly explain many issues related to AMR, such as its often-transient nature and aspects of its inheritance, there is a possibility that epigenetics may provide novel explanations and offer new insights into antimicrobial discovery.

## 2. Systematic Literature Review

**Figure 1:**
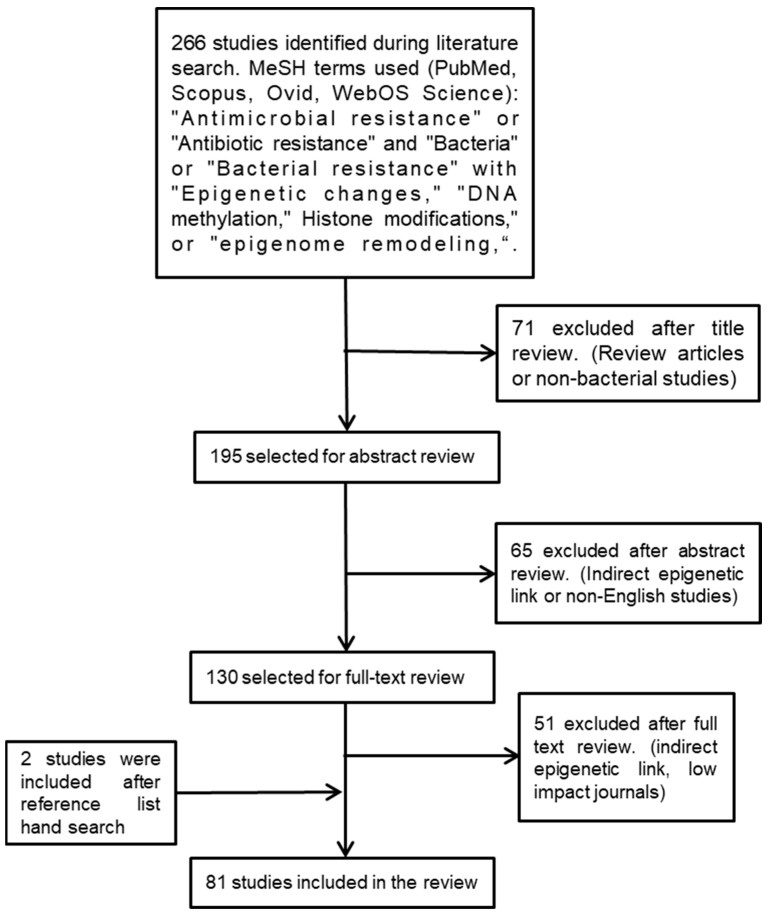
Flowchart outlining the strategy employed to identify the relevant studies.

We used the following medical subject heading (MeSH) terminologies to search for relevant studies: “antimicrobial resistance” or “antibiotic resistance” and “bacteria” or “bacterial resistance” with “epigenetic changes“, “DNA methylation”, histone modifications”, or “epigenome remodeling”. We searched several databases—namely, SCOPUS, Ovid, PubMed, and Web of Science—for scientific studies published during the past twenty years. To identify additional studies, reference lists of the selected studies were searched manually (Figure 1).

## 3. Eukaryotic Epigenetic Mechanisms

Epigenetics, in general, is a study of how environment and genes interact to modulate phenotypic changes via differential regulation of gene expression without any alteration in the DNA sequence. Epigenetic regulation includes three highly integrated mechanisms—DNA methylation, histone modifications, and regulation by noncoding RNAs (ncRNAs) [10]. This section briefly describes major epigenetic mechanisms involved in transcription regulation in eukaryotic cells.

### 3.1. DNA Methylation

DNA methylation is a fundamental DNA modification process that occurs mainly at the C5 position of cytosine residues (5mC) on DNA nucleotides and predominantly targets the CpG islands (CGIs). Of the whole genome, CGIs existing in a gene promoter region are most commonly subject to dynamic methylation modifications and gene regulation. DNA methyltransferases (DNMTs) are known as a family of enzymes that catalyze the process of DNA methylation, leading to gene silencing [11]. Additionally, a family of iron-dependent oxygenases—the ten-eleven translocation proteins (TETs)—function to remove the methyl group from the cytosine of the methylated DNA [12].

### 3.2. Histone Modifications

DNA is wrapped around core histone proteins, and these globular proteins have flexible tails protruding out from the nucleosome. Histone protein tails are subject to various post-translational covalent modifications, such as methylation, acetylation, and phosphorylation [13]. Histone acetyltransferases and deacetylases regulate the histone acetylation system. Histone deacetylation is usually associated with closed chromatin conformation and suppressing gene expression, whereas its acetylation will cause open chromatin conformation, increasing gene transcription [14]. On the other hand, histone methylation via arginine or lysine methyltransferases can facilitate or inhibit gene expression by regulating the DNA accessibility of transcription factors, gene silencing by blocking transcription, or gene overexpression by enabling the binding of transcription factors [15].

### 3.3. ncRNAs

There are various types of ncRNAs: The housekeeping ncRNAs include transfer RNA (tRNA), ribosomal RNA (rRNA), and small nuclear RNAs (snRNAs), while the regulatory ncRNAs include miRNA and lncRNA [16]. Multiple mRNAs can be targeted by miRNAs binding with the 3′-untranslated regions of mRNAs, leading to inhibition of protein expression. Likewise, lncRNAs modulate chromatin-modifying complexes or directly interact with transcription factors to suppress translation [17]. In recent years, many studies have shown that these ncRNAs play significant roles in epigenetic modification by targeting specific gene sequences and transposons, where they exert upregulation or silencing of the gene expression to control cell differentiation [18,19].

## 4. Overview of Bacterial Epigenetics

### 4.1. Bacterial DNA Methylation

Bacterial DNA methylation has been studied extensively (Table 1) [20]. The DNMTs present in bacteria are more commonly referred to as Mtases that are associated with the bacterial genome defense system, i.e., the restriction–modification (R–M) system. Additionally, a different class of Mtases exists without being associated with any endonucleases—the orphan Mtases—which have housekeeping functions. These Mtases transfer methyl groups to adenine and cytosine to specific genome sequences, leaving the unmethylated DNA sequence degraded by the R–M system [20]. In addition, several Mtases in the R–M system have been shown to have functions in phenotypic cell variations via regulation of transcription [21]. R–M systems represent one of the mechanisms by which bacteria protect themselves against exogenous DNA [22]. Mtases associated with the R–M system are abundantly found in the bacterial genome, the best example of which is *H. pylori*, whose genome encodes for more than 50 R–M-system-related Mtases [23]. 

The orphan Mtases are known to regulate bacterial growth by modulating the cell cycle, DNA mismatch repair, and gene expression [24]. These Mtases generally function as processive enzymes and methylate multiple targets by consecutive reactions without releasing their substrate DNA strand [25]. Deoxyadenosine methylase (Dam), found in *E. coli*, is an excellent example of an orphan Mtase that methylates the N^6^ position of the adenine residue, explicitly targeting the GATC sequence and playing a pivotal role in mismatch repair [26]. A common Dam-based methylation system involving GATC motifs, and several type-I R–M systems were identified across seven *K. pneumoniae* isolates [27]. Dam-mediated DNA methylation is also essential for regulating the cell cycle, gene expression, and transgenerational phase variation [28]. In addition to *E. coli*, homologs of Dam have been found in several other Gram-negative bacteria such as *Salmonella enterica* and *Vibrio cholera* [29]. Orphan Mtases are also known to methylate cytosine residues in growth-related genes. For instance, Dcm, found in *E. coli*, and VchM, found in *V. cholera*, control the expression of major gene regulators in the stationary growth phase but are not essential for bacterial survival [30]. Other types of well-studied orphan Mtases include Yhdj and CcrM; both of these Mtases methylate adenine residues at different locations and target different DNA sequences. CcrM is mainly reported to target hemimethylated DNA and regulates the bacterial cell cycle, mainly in Alphaproteobacteria [31]. 

### 4.2. Bacterial RNA Modifications

In addition to DNA modification, the presence of RNA modifications in bacterial rRNA, tRNA, and mRNA, depends on the bacterial growth cycle [32]. Of those, N6-methyladenosine (m6A) modification and 5′ NAD capping of mRNA have been reported as the most frequent type of modification in a wide range of bacteria, although the functional significance of RNA-modification-based epigenetic changes is unclear [33,34] (Table 1).

### 4.3. Bacterial Histone-like Proteins (HU)

**Figure 2:**
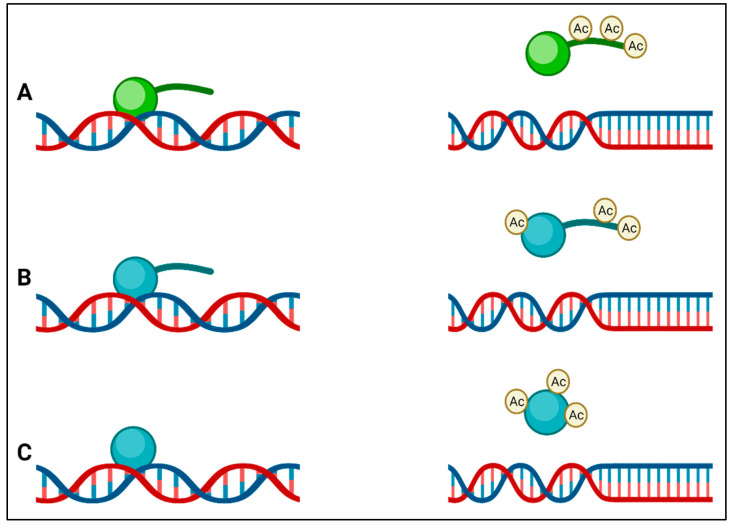
(**A**) Eukaryotic histones (green) have lysine-rich tails that are acetylated by lysine acetyltransferases, and this result in a reduction in affinity of the histone for DNA; (**B**) the histone-like protein (HU) (blue) of Mycobacterium also has a tail that is rich in lysines, which is acetylated by Eis, leading to a reduction in DNA affinity; (**C**) other bacterial HUs do not have tails but are acetylated at other positions to reduce their affinity to DNA.

Instead of having a membrane-bound nucleus similar to the nucleus of eukaryotes, bacteria pack their genomes into nucleoids through a series of nucleoid-associated proteins (NAPs) in distinct cytoplasmic regions. Differences in these NAPs are believed to form regions of chromatin, analogous to eukaryotic transcriptionally active heterochromatin and transcriptionally inactive euchromatin in bacteria [35]. Although it was previously claimed that bacteria do not possess histones and that bacterial epigenetics is limited to DNA methylation [20,36], there is now clear evidence that this is not the case. The HU in bacteria is a highly conserved low-molecular-weight NAP and is typically the most abundant across the bacterial kingdom, producing as many as 55,000 HU protein copies per cell in *E. coli* [37]. HUs have been called histone-like proteins due to the manner in which they bind DNA, and like eukaryotic histones, some bacteria (e.g., Mycobacterium and Campylobacter) have HUs with a lysine-rich C-terminal tail. The function of HU protein as a DNA-binding transcription factor indicates its influence on important metabolic processes such as initiation of DNA replication, induction of gene expression related to cell division, and stress response [38]. It can also be presumed to be involved in virulence gene expression in the case of pathogenic bacteria. In many bacteria, it has been found that modification of lysine residues by acetylation occurs on lysines within the core or the C-terminal tail that regulates DNA binding, (Figure 2) leading to the suggestion of an epigenetic histone-like code operating in bacteria [39]. The first evidence for these histone-like epigenetic changes came from a study using *Mycobacterium smegmatis*, in which heritable but semi-stable drug resistance was seen in bacterial subpopulations, which was determined to be due to the HU acetylation state [40]. Some of the enzymes that catalyze the acetylation of HU also acetylate aminoglycoside antibiotics, leading to their inactivation, and are important mediators of AMR [41]. HU-like histones usually act as transcription repressors, and in many bacteria, they are involved in the regulation of virulence and survival (Table 2).

## 5. Bacterial Epigenetics Causing Antibiotic Resistance

DNA methylation induced by Mtases by directly modulating the binding of RNA polymerases can cause positive and negative gene expression. The methylation of cytosine is mainly considered repressive and is commonly found in many pathogens. Such Mtase-mediated repressive feedback in the R–M system prevents methylation of phage DNAs when present inside the host, thus indirectly contributing to the development and promotion of antibiotic resistance [29]. Several lines of evidence, discussed below, support the notion that epigenetic mechanisms regulate the development of antibiotic resistance in bacteria, which are not fully explained by genetic changes alone.

The bacteria growing in subinhibitory concentrations of the antibiotics are known to develop adaptive resistance due to epigenetic changes. Thus, shifting the same bacteria to antibiotic-free media or exposure to a different type of antibiotic reverses the resistance effect. Therefore, the rapidity and reversible nature of such context-dependent AMR can only be explained by the appearance of epigenetic tags on the bacterial genome and not by genetic mutations [49]. However, only a few evolutionary and gene-knockout studies have identified epigenetic changes responsible for the development of adaptive resistance, but the role of Mtase-mediated epigenetic tagging of gene promoters influencing the binding of RNA polymerase might be the critical factor in the regulation of AMR-related gene expressions. For instance, in *E. coli*, Dcm-mediated DNA methylation induces the silencing of many genes encoding for ribosomal proteins [30].

Phase variation is a phenomenon where the bacteria can reversibly switch on or switch off specific genes to evade antibiotic effects. One way bacteria modulate the genes related to phase variation is via DNA hypermethylation or hypomethylation. Several Mtases exhibiting the function of phase-variable mediators have been found in bacteria. For instance, the expressions of LPS O-antigen in *S. enterica* and pap operon in *E. coli*, providing resistance via phase variation, are controlled by DNA methylation [50,51]. In *S. pneumoniae*, genetic rearrangement due to random gene switching leads to whole-genome methylation changes and phenotypic phase variation [52]. In *N. meningitidis*, adenine Mtases (ModA11, and ModA112) are known to increase susceptibility to certain antibiotics, which is strangely an evolutionary disadvantage. Nevertheless, the absence of these Mod proteins will increase the chances of bacterial survival [53]. Moreover, various Mtases demonstrating phase-variable expression have been discovered in *H. pylori* [54] and *Haemophilus influenzae* [55], supporting the role of epigenetic-mediated phase variation and development of AMR in these bacteria.

Phenotypic heterogeneity of bacterial population in a changing antibiotic milieu has been shown to induce heteroresistance and bistability, i.e., the appearance of two distinct bacterial subpopulations—the persister bacteria and the sensitive bacteria [56]. Persistent bacterial subpopulations can survive antibiotic treatment, but their growth will be slower or cell-cycle arrest will occur; however, after antibiotic withdrawal, these bacteria can relapse and cause reinfection. Several genetic-based mechanisms cause the survival of persistent bacteria, but recently, epigenetic inheritance has been reported as a potential contributor to the development of such phenotypes [57]. The appearance of heterogeneity and AMR phenotypes leading to recurrent infections has been reported in both Gram-negative and Gram-positive bacteria [58]. 

The transfer of antibiotic resistance genes in plasmids is known as plasmid-mediated resistance (PMR). This process occurs either via conjugation, with the help of bacteriophage viruses, or when some bacteria can pick up naked plasmids from the environment, and then those plasmids can be transferred between bacteria within the same species or between species. Plasmids frequently include several antibiotic resistance genes, which contribute to MDR’s spread. Antibiotic resistance mediated by MDR plasmids significantly limits treatment choices for bacterial infections, particularly in critically ill patients [59]. A rich variety of plasmids that can harbor numerous virulence factors and resistance genes exists in *K. pneumoniae*, which is the causative agent of serious community- and hospital-acquired infections [60]. Furthermore, there is also a potential epigenetic role of phage-encoded Mtases in AMR development. A vast portion (~20%) of the bacterial genome consists of genes that encode Mtases, which are incorporated into their genome via bacteriophages [61]. Such amalgamation of phage DNAs and bacterial genomes enhances the capability of bacteria to infect several different hosts. More than 800 different types of orphan Mtases were found to be encoded via bacteriophage DNA. For instance, the adenine methyltransferases encoded via phage DNA will methylate a specific DNA sequence, leading to packaging and protection of bacterial DNA from host restriction endonucleases and increasing bacterial survival [62]. However, more studies are needed to understand its application in antibiotic resistance. 

Lastly, in several species of bacteria, epigenetic processes contribute to developing AMR by regulating the genes not directly related to antibiotic resistance. For example, resistant strains of *M. tuberculosis* treated with 4-aminosalicylic acid showed differential methylation profiles in thousands of genes, mainly related to the ATP-binding cassette transporter proteins, ribosomal biogenesis pathway, and nitrogen metabolism pathway [63,64]. Integration of transcriptomic and epigenomic analysis in bacteria surviving under antibiotic stress can identify novel genes as potential targets and valuable assets to understanding indirect, epigenetic-mediated regulation of AMR. Nonetheless, our understanding of bacterial epigenetics and its role in antibiotic resistance development is still not fully understood. Moreover, pathogen-mediated host epigenome remodeling can also likely facilitate bacterial survival.

## 6. Bacteria-Induced Remodeling of the Host Epigenome

**Figure 3:**
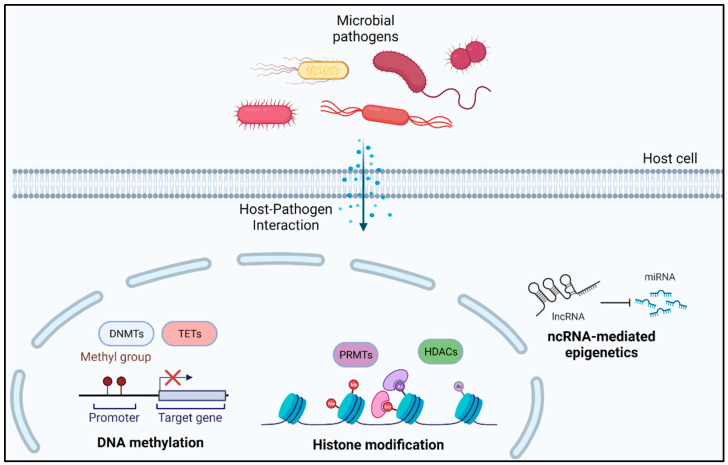
Bacteria-mediated epigenetic alterations in human host cells.

Bacterial-induced differential epigenetic changes in host cells can guide us toward the mechanism of development of immune tolerance and reduced immune response against invading pathogens [65]. In this section, we discuss various studies presenting evidence of bacteria-mediated epigenetic alterations in human host cells, indirectly facilitating bacterial survival (Figure 3).

### 6.1. Human Host DNA Methylation

*H. pylori* is one of the most commonly found bacteria in the human stomach and can survive for a long time in many patients, even without producing any symptoms or causing severe types of gastric disorders, such as peptic ulcers, chronic gastritis, gastric adenocarcinoma, and mucosa-associated lymphoma, in some individuals [66]. Studies have reported the ability of *H. pylori* in changing the epigenome of host gastric epithelial cells [67]. Mainly, these changes are *H. pylori* virulence-factor-induced DNA-methylation-based epigenetic regulation of gene expression in gastric epithelial cells [68,69]. As a downstream effect, *H. pylori* directly or indirectly manipulate carcinogenic transformation pathways, facilitating cancer development [69]. Additionally, it has been reported that acute *H. pylori* infection stimulates the production of endogenous antimicrobial peptides from gastric epithelial cells [70,71]. Later, it was revealed that a chronic infection probably suppresses the EGFR-mediated pathways associated with the release of human beta-defensin of the innate antimicrobial defense system leading to persistent infection [71,72]. However, our knowledge of the role of epigenetic effects mediated by *H. pylori* infection in supporting resistance against antibiotics is limited, and the only clear link is the presence of R–M-system-associated DNA methyltransferases exhibiting phase variation mechanisms [73]. 

Similarly, *M. tuberculosis*, the causative agent of tuberculosis (TB), has been reported to induce DNA methylation in host cells. TB is highly prevalent in developing countries. The WHO Global Tuberculosis 2021 Report stated that TB is the 13th leading cause of death and the 2nd leading infectious killer. MDR-TB remains a public health crisis, and only about one in three people have access to its treatment [74]. The appearance of antibiotic-resistant *M. tuberculosis* is on the rise, posing a significant threat to public health [75]. TB resistance to multiple drugs is associated with a relatively poor treatment success rate, and the unique nature of the bacteria cell wall is considered an intrinsic contributor [76]. A study of human monocytic dendritic cells infected with *M. tuberculosis* showed DNA methylation of distal enhancer gene elements, which regulates the activation of key immune transcription factors [77]. In addition, *M. tuberculosis* also induces methylation of non-CpG islands, suggesting a role in global methylation changes supporting bacterial pathogenesis [78]. Additionally, *M. tuberculosis* infection has been reported to cause methylome changes in genes involved in T-cell responses, cytoskeleton organization, and cytokine production [79]. Overall, these epigenetic tags can be modulated to regulate the mechanisms required to develop more robust transcriptional responses upon reinfection and decreased resistance to secondary infection [80].

In addition, there are many other bacteria that were reported to induce DNA methylation in eukaryotic host cells via upregulation of DNMTs, causing silencing of tumor suppressor genes, such as the downregulation of cyclin-dependent kinase inhibitor 2A (*CDKN2A*) in uroepithelial cells via *E. coli* infection, allowing them to persist and proliferate [80]. Macrophages infected with *Burkholderia pseudomallei* induce significant DNA methylation throughout the whole genome—namely, in the promoter regions of the genes involved in inflammatory responses and cell survival [79]. Lastly, it has also been suggested that bacterial infection can induce transgenerational epigenomic reprogramming. For instance, the *Campylobacter rectus* infection in pregnant mothers induces hypermethylation repression of insulin-like growth factor gene in the fetus, causing poor growth and developmental abnormalities [81].

### 6.2. Histone Modifications

Bacteria can directly or indirectly manipulate histone tags or modulate the acetyltransferase enzyme system to induce histone acetylation in host cells. Usually, bacteria have adapted mechanisms to use this acetylation system for their benefit by specifically suppressing the inflammatory response genes or inhibiting the immune response-related genes in general [82]. *Listeria monocytogenes* is a Gram-positive facultative anaerobic bacterium that causes listeriosis, a systemic infectious disease primarily affecting pregnant women and leading to spontaneous abortion [83]. MAPK-mediated H4 acetylation in listeriosis leads to the upregulation of many interleukin-related genes and increased accumulation of neutrophils at the site of infection [84]. *L. monocytogenes*-mediated deacetylation of H3K18 also plays a critical role in reprogramming the host response [85]. Furthermore, nosocomial infection, caused by *P. aeruginosa*, may possess an intrinsic mechanism to induce global hypoacetylation in H3K18 or upregulation of histone deacetylase (HDAC), leading to downregulation of TNF, interleukins, and chemokines, making it resistant to many classes of antimicrobial therapy [86]. *Legionella pneumophila*, a Gram-negative bacterium, secretes methyltransferase-catalyzing histone H3K14 trimethylation, leading to downregulation of Toll-like receptors and interleukins suppressing innate immune response in human monocytes and alveolar epithelial cells [87]. Moreover, bacterial LPS has been shown to induce immune paralysis via histone modification, leading to a high risk of critical illness upon reinfection. In addition to LPS, bacteria can modulate epigenetic marks on histones via the direct action of secreted metabolites [88]. Epigenomic profiling of histone modifications in severe bacterial infections will provide a way forward in understanding the mechanisms of AMR in clinically relevant infectious diseases.

### 6.3. ncRNA-Mediated Epigenetic Modifications

MicroRNA (miRNA) is a type of ncRNA that can epigenetically mediate mRNA translation. Cellular overexpression causing an increase in the plasma levels of miRNA has been reported in *M. tuberculosis* infection. TB patients, when compared with healthy controls, showed higher levels of circulating miRNAs (miR-361-5p, miR-484, miR-425, miR-769-5p, miR-769-5p, miR-320a, and miR-22-3p). In clinical settings, overexpression of these miRNAs can suggest treatment failure probably due to drug resistance, although no specific antibiotics were mentioned, and there is a possibility that different antibiotics can trigger different mechanisms of epigenetic modification. However, the contrasting levels of these miRNAs can be utilized to classify patients into responders versus nonresponders, thus having the potential to be used as diagnostic biomarkers [89,90,91]. A study also reported differential expression of over 700 lncRNAs in blood mononuclear cells in response to drug-resistant TB infection, and some lncRNAs were associated with regulating host immune response against the infection [92]. They confirmed the role of bacteria modulating the host genome via regulating the expression of ncRNAs.

Moreover, a bacterial infection of injured skin is associated with delayed healing and poor treatment outcome. LPS induces chronic inflammation and upregulates endoplasmic reticulum (ER) stress, to directly interfere with cytokine signaling by reducing STAT3 phosphorylation, thereby inhibiting the expression of SOCS3 [93]. It has been shown that bacteria secreted LPS induces downregulation of miR-211-3p in skin fibroblast cells to activate ER stress-related molecules and reduce cell proliferation in these cells, suggesting delayed wound healing post-infection [94]. Characterization of ncRNAs pairing with mRNAs and the underlying pathological mechanisms may help understand the factors involved in determining the ncRNA–mRNA specificity and the impact of introducing these ncRNAs into the host cell for the development of RNA-based antimicrobial strategies to fight MDR infection [95].

Overall, these findings suggest that pathogens following infection can induce specific and generalized epigenetic changes. All these epigenetic changes somehow support microbial survival or enhance disease outcomes. A better understanding of host epigenetic alteration upon long-term bacterial infection allows us to use “epigenetic changes targeting drugs” as newer antibiotics, thus encountering the problem of therapeutic resistance.

## 7. Potentials of Using Epigenetic Drugs as Newer Antimicrobial Agents

Epigenetic drugs, also known as “epidrugs”, are small molecules that can modulate gene expression by either targeting bacterial as well as the host DNA methyltransferases or chromatin modifiers. Moreover, the use of a poly-pharmacological approach to target numerous chromatin-modifying epigenetic enzymes may constitute a smarter option [96]. Given that bacteria package their DNA differently than humans, many enzymes that induce epigenetic changes targeting human cells do not exist in bacteria. However, evidence exists that direct inhibition of epigenetic enzymes can alter the bacterial genome affecting its survival. For example, epigenetic modulator UVI5008, having anti-gyrase activity, causes disruption of the cell wall in MDR *S. aureus*, leading to the reversal of antibiotic resistance of a previously resistant drug [97]. A major component of green tea, epigallocatechin-3-gallate (EGCG), is a potential epigenetic modifier, which has been reported to alter DNA methylation and also demonstrated synergistic antimicrobial potential against antibiotic-resistant nosocomial *S. aureus* [98,99]. Overexpression of Dam in *V. cholera* and *Yersinia pseudotuberculosis* diminished their virulence, eliciting a strong host immune response [100]. Moreover, the knockdown of three R–M systems in *E. coli* showed no effect on antibiotic susceptibility or host immune response against the pathogen [101]. 

HDAC inhibitors (HDACis) have been shown to exert antiviral effects; thus, several studies have reported the use of HDACis in combination with oncolytic virotherapy to reduce the viral reservoir for cancer patients (HIV) [102,103]. HADCis can block substrate binding on the active site of HDAC enzymes to suppress their activity [95]. In host cells, HDACi treatment can increase the expression of effectors of the innate immune response against bacterial infections [82]. For example, when *E. coli*-infected colon epithelial cells were treated with trichostatin A, an HDACi, it upregulated the expression of the *HBD2* gene and suppressed the release of inflammatory cytokine IL-8, preventing the tissue damage associated with excessive inflammation [104]. Furthermore, HDACis affect several other aspects of the host immune response relevant to MDR development in bacteria. Long-term HDACi treatment might compromise host defense, and selective HDAC inhibitors have successfully treated acute bacterial infections. For example, co-treatment with tubastatin A, an HDAC6 inhibitor, induced mitochondrial reactive oxygen free radicals and facilitated clearance of *S. Typhimurium* and *E. coli* from human macrophages by enhancing phagocytosis [105]. 

Furthermore, with the characterization of novel ncRNAs in bacteria, targeting these ncRNA molecules might provide a promising therapeutic application against pathogens. For instance, custom ncRNA cassettes carrying the antisense sequence of a target mRNA were delivered to different *E. coli* strains, which helped control bacterial gene expression [106]. Another example, *S. enteritica*, has been reported to upregulate miR-128 expression in intestinal epithelial cells, depressing the macrophage recruitment at the site of infection. The secreted proteins from *S. enteritica* activated the p53 signaling pathway, inducing miR-128 upregulation, and treatment with anti-miR-128 showed a significant increase in macrophage recruitment suppressing the infection load [107]. These findings suggest that epigenetic-process-mediating medications might be a promising treatment strategy for treating MDR infections in a patient suffering from antibiotic resistance.

## 8. Conclusions and Future Directions

Since well-documented biochemical or genetic alterations are unable to explain the processes driving antibiotic resistance adequately, researchers must turn their attention to newer, nonclassical mechanisms such as epigenetic control. It is becoming clear that bacteria possess sophisticated epigenetic regulatory mechanisms, with many similarities to eukaryotic epigenetics. Epigenetics now explains various hitherto puzzling bacterial phenomena, such as switching or phase variation in gene expression and persistent bacteria that exhibit resistance to lethal concentrations of antibiotics. However, our present understanding of bacterial epigenetics is still far from complete. Beyond R–M systems, for example, the significance of methylation in bacterial genomes is not well-known. The importance of understanding the role of epigenetic processes in bacterial antibiotic resistance, genome- and transcriptome-wide probing, and functional assessments of differentially methylated genes or regulatory elements is becoming increasingly apparent.

The discovery of epigenetic mechanisms in pathogenic bacteria reveals new targets for the development of novel antibiotics, and this has already begun. The HU acetylation enzyme Eis has been targeted by haloperidol analogs, and although problems remain with the neurotoxicity of these compounds, it is hoped that related non-neurotoxic analogs may be found to be useful against *M. tuberculosis* [108]. Many of the bacterial NAPs do not appear to be homologous to eukaryotic proteins, so they may also present fresh targets for new antibiotics. Additionally, current sequencing methods allow for single-base resolution of bacterial transcriptomes, permitting direct detection of mRNA nucleotide changes. While the literature reviewed in this study (summarized in Supplementary Table S1) clearly suggests that epigenetics has a role in bacterial antibiotic resistance, the accurate identification of epigenetic labels on bacterial genomes and their functional characterization will help achieve a significant leap in our understanding of this field. Moreover, epigenetic changes in bacterial genomes might serve as new diagnostic markers and therapeutic targets.

Innate immunity is mediated by pattern recognition receptors that recognize pathogen-associated microbial patterns and mediate an inducible response to microorganisms. These interactions between specific bacterial motifs and the innate immune system are crucial in determining an individual’s vulnerability to severe diseases [109]. However, the mechanisms regulating these molecular networks during various stages of infection are still a mystery. In patients suffering from MDR bacterial-infection-associated critical illnesses, advanced next-generation sequencing (NGS) platforms and bioinformatic analysis can strongly support the bacterial remodeling of host epigenomes. In data obtained from such NGS epigenomic studies, the key is to identify microbe-specific sites likely to be affected by aberrant DNA methylation, histone modifications, and noncoding RNA induction. Indeed, successful therapeutic strategies that are able to manipulate the innate immune memory of infectious diseases will emerge from a comprehensive understanding of key molecular networks and epigenetic interactions. Instead of a single mutation, a network medicine method combined with transgenerational effects may reveal a chain of epigenetic modifications perturbing the human interactome across time and between generations. There is still a wide debate about the epigenome modification of the host by pathogens, and a substantial number of clinical studies should be conducted to see if these findings can be transferred from the laboratory to critically ill patients.

## Figures and Tables

**Table 1:** Overview of bacterial epigenetics through DNA and RNA modifications.

Modifications	Types	Enzymatic Systems	Functions	Examples
DNA	Methylation	R–M system	Defense mechanism	*EcoRV*, *CfrBI*
		Orphan Mtases	Adenine and Cytosine methyltransferases cause regulation of cell cycle, DNA repair, and gene expression	*DAM*, *Dcm*, *CcrM*, *YhdJ*, *VchM*
	Phosphorothioation	DNA degradation	Defense mechanism	*dndABCDE*
RNA	Methylation	N^6^-methyladenine modifications	ND ^1^	ND ^1^
	Capping	5′ NAD capping	Prevent RNA degradation	ND ^1^

^1^ Not determined.

**Table 2:** The epigenetic regulation of pathogenic effectors through HU proteins.

Bacterial Species	HU	Details	Reference
*Burkholderia cepacia*	Bmul_0158	Fourfold upregulation of Bmul_0158 is associated with several virulence traits.	[42]
*E. coli* K-12	HUαE38K, V42L	Transforms to an invasive phenotype and replicates in host cells by escaping from phagosomal and by subversion of host cell apoptosis.	[43]
*Bacillus subtilis*	HBsu	Ensures chromatin packing during sporulation.	[44]
*Acinetobacter baumannii*	H-NS	Regulates antibiotic resistance.	[45]
*Vibrio cholerae*	H-NS	Regulates virulence, the stress response, and chemotaxis.	[46]
*Shigella* spp.	H-NS	Regulates intra-cellular invasiveness.	[47]
*Xanthomonas citri*	HupB	Regulates flagellar development and biofilm production.	[48]

## Data Availability

Not applicable.

## Supplementary Materials

The following are available online at https://www.mdpi.com/article/10.3390/antibiotics11060809/s1, Table S1: Epigenetic mechanisms and the related articles reviewed in this study.
