# Supplementary material for: Epigenetic-Mediated Antimicrobial Resistance: Host versus Pathogen Epigenetic Alterations
Source: Antibiotics (Basel). 2022 Jun 16;11(6):809. doi: 10.3390/antibiotics11060809 (PMC9220109; doi:10.3390/antibiotics11060809)
Supplement: Supplementary file 1 [file antibiotics-11-00809-s001.zip › antibiotics-1705847-supplementary.pdf]

Supplementary Table S1: Epigenetic mechanisms and the related articles reviewed in this study.

	**Epigenetic mechanism(s)**	**References**
Bacterial epigenetics	DNA methylation	[20-31, 50-58, 63, 64]
	RNA modification	[32-34]
	Histone-like proteins	[35-37, 39-48, 108]
	Plasmid-mediated epigenetics	[59-62]
Human host epigenetics	DNA methylation	[65, 67-73, 77-81]
	Histone modification	[82-88]
	ncRNA-mediated modifications	[89-92, 95]
Epidrugs	DNA methylation	[97-101]
	Histone modification	[82, 95, 102-105]
	RNA modification	[106, 107]
